# Atosiban application in fresh ET cycle is effective for women undergoing repeated embryo implantation failures, especially for advanced-age obese patients

**DOI:** 10.1038/s41598-023-49773-z

**Published:** 2023-12-27

**Authors:** Jie Li, Sien Mo, Zhong Lin, Qiuling Shi

**Affiliations:** 1 State Key Laboratory of Ultrasound in Medicine and Engineering, College of Biomedical Engineering, Chongqing Medical University, Chongqing, 400016 China; 2 The Reproductive Hospital of Guangxi Zhuang Autonomous Region, Nanning, China; 3 School of Public Health, Chongqing Medical University, Chongqing, China

**Keywords:** Epidemiology, Obesity, Infertility

## Abstract

To assess the effect of atosiban in pregnancy outcomes of the fresh embryo transfer (ET), the retrospective cohort study was conducted. Six hundred and eighty-nine cases (using atosiban) and 1377 age and ET cycle-matched controls were collected from the January 2016 to May 2022 to perform the fresh IVF-ET cycle. The essential characteristics and pregnancy outcomes were analyzed. Conditional logistic regression analysis and subgroup analysis were performed. In the whole samples, atosiban had no effects in the pregnancy outcomes. Subgroup analyses suggested that atosiban could improve the clinical pregnancy in more than 3 ET cycles (OR 1.667, 95% CI 1.108–2.509, *P* = 0.014). Moreover, the improvement of clinical pregnancy was mainly present in the advanced-age women (age ≥ 35 years: OR 1.851, 95% CI 1.136–3.014, *P* = 0.013), obesity (BMI ≥ 24 kg/m^2^: OR 2.550, 95% CI 1.105–5.883, *P* = 0.028) and cleavage stage embryo (D3 embryo: OR 1.721, 95% CI 1.098–2.696, *P* = 0.018) among the repeated implantation failures (RIF). Atosiban could also improve the live birth for the obese women. Further, in the RIF, atosiban application was strongly recommended for the advanced-age infertility women, who also had the risk of obesity with the implantation of the cleavage stage embryo. In conclusion, atosiban could improve pregnancy outcomes for the advanced-age and obese women in RIF, especially while implanting the cleavage stage embryo in fresh ET cycle.

## Introduction

Infertility is a serious public health issue influencing about 8–12% couples^1^. As an important treatment of infertility, in vitro fertilization–embryo transfer (IVF-ET) has been applied widely. However, the successful rate of IVF-ET was estimated to be about 30%^2^. Many factors were identified to affect the outcome of IVF-ET, such as embryo quality, endometrial status, intrauterine environment, etc., in which the uterine contractions occurred in 30% patients in the process of IVF-ET^3,4^. Moreover, uterine contraction frequency remained notably four days after human chorionic gonadotropin (hCG) administration, even at the time of embryo transfer^4^. In the ET cycle, more frequent uterine contraction comparing to natural cycles, would lead to the poor pregnancy success^5^. As one of common type in the IVF, fresh embryo transfer cycle was performed widely with hyperstimulated ovulation induction drugs to recruit more oocytes. However, in this process, high estrogen (E_2_) levels would not only affect endometrial receptivity, but also induce uterine contractions more easily^6,7^. In general, when a serum E_2_ level > 3000–5000 pg/mL and/or the number of retrieved oocytes more than 15–20 in hyperstimulated IVF cycles, freezing all embryos is an effective method to decrease the occurrence of ovarian hyperstimulation syndrome. Comparing with the fresh embryos transferred in the hyperstimulated cycles, transferring these frozen-thawed embryos in subsequent cycles had been found to achieve higher pregnancy rates. Thus, the researchers had speculated that endometrial receptivity is impaired under the direct effect of high E_2_ concentrations including a greater propensity to induce uterine contractions^8–10^. In our center, to reduce the uterine contractions induced by E_2_ and decrease the risk of ovarian hyperstimulation, the E_2_ level was also restricted lower than 4000 pg/mL at the time of the oocyte retrieval for patients in fresh embryo transfer cycle. So, understanding and restraining the uterine contractions in fresh embryo transfer cycle would improve the pregnancy outcomes effectively.

Although approximately 30% of patients experienced increased uterine contractions at the time of IVF-ET, decreasing uterine contractions before IVF-ET presently lacks clear diagnostic measures and therapies^11,12^. Many studies attributed the uterine contraction to the production of oxytocin and prostaglandin (PG)F2a from endometrial cells^13,14^. Moreover, the application of PGF2a and oxytocin-antagonists may improve the pregnancy outcomes in practice^15^. As a mixed oxytocin/vasopressin V1a receptor antagonist, atosiban was used to reduce uterine contractility and improve IVF-ET success by interfering with the oxytocin/PGF2a system with minimal side effects^16,17^. In 2007, Pierzynski et al.^18,19^ reported the safety and effectiveness of atosiban in the IVF-ET. Then, a randomized, placebo-controlled clinical study also confirmed that the atosiban could improve the clinical pregnancy rate prominently^20^. Meta-analysis also identified the positive effect of atosiban in increasing the clinical pregnancy rates^21^. However, a randomized double-blind study had negated these conclusions, in which no significant effectiveness of atosiban was identified in the pregnancy success rate^22^. And these negative results would be attributed to the unassorted ET cycles. Our meta-analysis discovered that atosiban could improve the successful rate of IVF-ET among women undergoing third or more ET cycle^23^. Recent study also confirmed the conclusion that atosiban might be an important factor in enhancing pregnancy outcomes for the repeated embryo implantation failure^24^. In 2016, He et al.^25^ also suggested that the atosiban treatment would be effective in improving frozen-thawed embryo implantation from the third ET cycle. However, in 2022, Tang et al.^26^ had not discovered the effectiveness of atosiban, especially for the women with repeated implantation failures (RIF) receiving the fresh embryo transfer. Considering the inconsistent clinical role of atosiban for the fresh embryo ET, this retrospective cohort study was conducted to discuss the effects of atosiban in the fresh embryo implantation cycle specially. In order to ensure comparability between two groups, the E_2_ level did not have significant difference in Atosiban and Control groups on the day of hCG administration. The results would provide a reliable selection of medication for the clinicians, and bring more hope to patients with RIF especially for the fresh ET.

## Methods and materials

### Participant’s selection

A retrospective cohort study was conducted. Participants all came to the Reproductive Hospital of Guangxi Zhuang Autonomous Region from January 2016 to May 2022 to perform the fresh IVF-ET cycle. The essential information and follow-up data of the pregnancy outcomes were collected from our electronic medical record system. All included patients signed informed consent for IVF-ET and medication. The study was approved by the medical ethics committee of the Reproductive Hospital of Guangxi Zhuang Autonomous Region (No. KY-LL-2022-13), and performed in accordance with the Declaration of Helsinki.

### Inclusion and exclusion criteria

The patients included in our analysis met the inclusion criteria as follows: (1) with complete information of clinical pregnancy, age and embryo transfer cycle (2) the morphology of uterus was normal evaluated by the transvaginal ultrasound; (3) at least one high-quality embryo was transferred. Moreover, the patients would exclude according to the exclusion criteria: (1) endometriosis and adenomyoma of the uterus; (2) uterine anomaly or uterine fibroids; (3) endometrial thickness less than 7 mm; (4) diminished ovarian reserve (antral follicle counts ≤ 5–7 or anti-Mullerian Hormone ≤ 1.1 ng/mL); (5) hydrosalpinx without intervention; (6) endocrine disorders, such as hypothyroidism or hyperthyroidism, hyperprolactinaemia, or premature ovarian failure. Then, 689 cases using the atosiban were included in the cohort.

### Intervention vs. control matching

The use of atosiban followed the patient’s wishes, after informing patients of the possible effects, reactions and costs of atosiban. Controls were randomly selected from 15,838 patients who did not use the atosiban. The ages and embryo transfer cycles were used in the logistic regression model for the propensity score calculation and matching. Using a 1:2 matched intervention-control design with 0.02 caliper value was selected randomly in the propensity score matching (PSM). The adequate balances of matching were defined as the standardized mean differences between groups less than 10%^27^. Finally, 1377 controls were included in the following analysis.

### Interventions

In addition to the medications used routinely in our IVF program, the patients in the atosiban group received intravenous atosiban 30 min before ET with a bolus dose of 6.75 mg, within 1–2 min infusion time. After performing the ET procedure, the same dose of atosiban (6.75 mg, within 1–2 min infusion time) was continued within 30 min. Patients in the control group received treatment plan as similar as atosiban group, except for the atosiban.

### Medication protocol

All enrolled patients were treated with the gonadotrophin releasing hormone agonist (GnRH-a) protocol. The GnRH agonist was used for down-regulation at luteal phase including long-term GnRH-a 1/3–1/2 branch and short-term GnRH-a 0.05–0.1 mg/day. Check pituitary down regulation standards after 14 days: blood levels for E_2_ < 50 ng/L, FSH < 5 IU/L, LH < 5 IU/L, follicle diameter ≤ 5 mm, the thickness of endometrium ≤ 5 mm.

The starting dosage was 150–225 IU/day conventionally for ovarian hyperstimulation, which would be estimated comprehensively on the basis of the female age, basic FSH level, antral follicle count, BMI and the gonadotropin (Gn) dosage. Then, the follicular development and the blood E_2_, LH and Prog levels were monitored. According to these results, the Gn dosage was adjusted individually. Once, 18 mm follicle had developed, ovulation triggering was induced with 10,000 IU hCG. After 34–36 h, oocyte was retrieved place.

According to the blood beta hCG level after embryo transfer for 14 days, pregnancy was assessed. Once the patients were pregnant, vaginal ultrasound would be conducted in 28 days later. The gestational sac detected by vaginal ultrasound was defined as the clinical pregnancy. Then, continue to luteal support for 10–12 weeks of pregnancy.

### Hormonal measurements

The levels of six hormones including the follicle-stimulating hormone (FSH), estrogen (E_2_), progestogen (Prog), prolactin (PRL), luteinizing hormone (LH), and testosterone (T) were detected in the Department of Clinical Laboratory at the Reproductive Hospital of Guangxi Zhuang Autonomous Region with electrochemiluminescence immunoassays and immunoturbidimetric methods.

### Pregnancy outcomes and covariates

The pregnancy outcomes including the clinical pregnancy, ectopic pregnancy, abortion, and live birth were recorded. In addition to the main pregnancy outcomes, the essential characteristics (age, duration of infertility, embryo category, embryo transfer cycle, types of infertility, etc.) and physical examinations (height, weight, etc.) were also collected. The body mass index (BMI) was calculated according to the formula: body weight (kg)/height^2^ (m).

### Statistical analysis

The statistical differences were calculated with the Student's t test and Χ^2^ test for the continuous and categorical variables respectively. Then, the conditional regression and binary logistic regression were performed to evaluate the association between the atosiban application and pregnancy outcomes after adjusting the confounding factors in the pooled and subgroup analyses, respectively. Three adjusted models were applied in every regression analysis (unadjusted, duration of infertility-adjusted, and multivariate-adjusted models). The covariates in the multivariate-adjusted model included age, BMI, duration of infertility, embryo category, embryo transfer cycle, FSH, E_2_, P, PRL, LH, and T levels. Subgroup analysis was then conducted. On the basis of age, the participants were divided into advanced-age group (age ≥ 35 years) and non-advanced age group (age < 35 years). According to the Chinese standard, BMI was defined as normal weight (< 24.0 kg/m^2^) and overweight/obese (≥ 24.0 kg/m^2^)^28^. Moreover, the embryo category was defined as blastocyst stage (D5/D6) and cleavage stage (D3) embryos. Finally, cycles of previous embryo transfer failure were categorized as 1 cycle, 2 cycle and ≥ 3 ET cycles. SPSS version 24.0 software (SPSS Inc., Chicago, IL, USA) was used in all the analyses. The statistical tests were two-tailed, with 0.05 as the significant level.

### Ethical approval

The study was approved by the medical ethics committee of the Reproductive Hospital of Guangxi Zhuang Autonomous Region (No. KY-LL-2022-13).

### Consent to participate

Informed consent was obtained from all individual participants included in the study.

## Results

### Essential characteristics of the participants

In this study, 689 patients had received the atosiban plus routine treatment. And 15,838 patients received routine treatment only. Compared with those in the control group, patients using atosiban were older (*P* < 0.001), with lower E_2_ level (*P* = 0.025), and a higher embryo cycles (*P* < 0.001) (Table S1). In order to control the selection bias, 1377 matching controls were collected with the PSM. The standardized mean differences between groups were less than 10%, which indicated the adequate balances of matching. In the new cohort, the age, BMI, duration of infertility and the levels of FSH, E_2_, P, PRL, LH and T, all had no significant statistic differences (Table 1). Moreover, no statistic differences were also shown in the embryo category, embryo transfer cycle, types of infertility and pregnancy outcomes (Table 1). These consistencies guaranteed the reliability of the analyses.

**Table 1:** The characteristics of the eligible samples.

	Atosiban	Control	P
No	689	1377	
Age	35.58 ± 4.80	35.55 ± 4.85	0.914
BMI	22.35 ± 2.66	22.52 ± 2.81	0.190
Duration of infertility	4.83 ± 3.60	5.11 ± 3.76	0.110
Numbers of embryos transfer	1.82 ± 0.39	1.75 ± 0.43	< 0.001
FSH	7.88 ± 2.82	7.92 ± 2.78	0.785
E_2_	43.23 ± 26.30	45.52 ± 29.70	0.087
Progestogen	0.13 ± 1.70	0.06 ± 0.65	0.179
PRL	17.14 ± 12.62	16.27 ± 12.71	0.142
LH	5.14 ± 3.31	4.94 ± 2.56	0.138
T	0.47 ± 3.00	0.40 ± 1.25	0.475
Types of infertility			
Primary	201	387	
Secondary	488	990	0.612
Embryo category			
D3	528	1048	
D5/D6	161	329	0.791
Embryo transfer cycle			
1	224	488	
2	286	572	
≥3	179	357	0.680
Clinical pregnancy			
Yes	255	517	
No	434	860	0.813
Ectopic pregnancy			
Yes	9	20	
No	680	1357	1.000
Abortion^a^			
Yes	52	98	
No	615	1253	0.662
Live birth^a^			
Yes	172	373	
No	495	978	0.386

*BMI body mass index, *FSH* follicle-stimulating hormone, *E*_*2*_ estradiol, *P* progestogen, *PRL* prolactin, *LH* luteinizing hormone, *T* testosterone.

*^a^48 patients have loss to follow-up for the outcomes of abortion and live birth.

### Atosiban had no effects in the pregnancy outcomes in the whole samples

Among the total matched sample, no significant effects of atosiban in the pooled pregnancy outcomes were discovered. After adjusting the duration of infertility and multi-adjusted analyses for all participants, we still did not detect any statistical differences in pregnancy outcomes (Table 2). Furthermore, atosiban also had no impacts on pregnancy outcomes in the subgroup stratified by age, BMI, and embryo category, respectively (Tables S2, S3, S4).

**Table 2:** The effects of atosiban in the outcomes of pregnancy with cox regression analysis.

	Unadjusted				Duration of infertility-adjusted			Multi-Adjusted		
	OR	95% CI	*P*		OR	95% CI	*P*	OR	95% CI	*P*
Clinical pregnancy	0.986	0.848–1.145	0.849		0.983	0.846–1.142	0.822	1.001	0.860–1.166	0.987
Ectopic pregnancy	0.900	0.410–1.977	0.793		0.896	0.406–1.977	0.786	1.045	0.427–2.560	0.923
Abortion	1.073	0.765–1.504	0.684		1.072	0.765–1.504	0.686	1.185	0.818–1.717	0.370
Live birth	0.928	0.774–1.113	0.419		0.917	0.764–1.100	0.352	0.951	0.788–1.149	0.605

*Multi-adjusted: age, BMI, duration of infertility, embryo category, FSH, E_2_, P, PRL, LH, and T levels.

*OR odd ratio, *CI* confidence interval, *BMI* body mass index, *FSH* follicle-stimulating hormone, *E*_*2*_ estradiol, *P* progestogen, *PRL* prolactin, *LH* luteinizing hormone, *T* testosterone.

### Atosiban improved the clinical pregnancy outcomes in more than 3 ET cycles

As shown in results, advanced age, obesity and embryo category could not influence the functions of atosiban in the pregnancy success independently (Tables S2, S3, S4). Oppositely, embryo transfer cycles might significantly disturb the effects of atosiban in the IVF-ET (Table 3). Although clinical pregnancy and live birth in the 1 ET cycle were benefited from atosiban, the correlations were disappeared after adjusting the confounding factor (Table 3). Only in more than 3 ET cycles, atosiban could improve the successful rate of clinical pregnancy steadily (unadjusted: OR 1.685, 95% CI 1.141–2.491, *P* = 0.009; duration of infertility-adjusted: OR 1.685, 95% CI 1.140–2.492, *P* = 0.009; multi-adjusted: OR 1.667, 95% CI 1.108–2.509, *P* = 0.014) (Table 3). However, atosiban might not influence the other pregnancy outcomes (ectopic pregnancy, abortion and live birth) (Table 3).

**Table 3:** The effects of atosiban in the outcomes of pregnancy on the basis of the embryo cycles.

	Unadjusted			Duration of infertility-adjusted			Multi-adjusted		
	OR	95% CI	P	OR	95% CI	P	OR	95% CI	P
Cycle 1									
Clinical pregnancy	0.703	0.508–0.972	0.033	0.687	0.496–0.952	0.024	0.753	0.537–1.056	0.100
Ectopic pregnancy	0.764	0.269–2.170	0.613	0.739	0.259–2.107	0.572	0.911	0.311–2.673	0.865
Abortion	1.116	0.638–1.952	0.700	1.153	0.657–2.024	0.620	0.985	0.548–1.770	0.960
Live birth	0.666	0.470–0.943	0.022	0.645	0.454–0.915	0.014	0.729	0.505–1.051	0.091
Cycle 2									
Clinical pregnancy	0.948	0.705–1.275	0.725	0.949	0.705–1.276	0.728	0.903	0.665–1.226	0.514
Ectopic pregnancy	0.799	0.154–4.142	0.789	0.781	0.150–4.060	0.768	0.884	0.159–4.926	0.888
Abortion	0.964	0.530–1.753	0.904	0.971	0.533–1.766	0.922	0.986	0.533–1.824	0.963
Live birth	0.975	0.704–1.349	0.877	0.974	0.704–1.349	0.876	0.891	0.636–1.248	0.501
Cycle ≥ 3									
Clinical pregnancy	1.685	1.141–2.491	0.009	1.685	1.140–2.492	0.009	1.667	1.108–2.509	0.014
Ectopic pregnancy	2.006	0.280–14.357	0.488	2.078	0.289–14.933	0.467	1.687	0.201–14.177	0.630
Abortion	1.205	0.609–2.383	0.592	1.208	0.610–2.392	0.588	1.272	0.630–2.569	0.503
Live birth	1.391	0.866–2.235	0.173	1.388	0.863–2.232	0.176	1.313	0.795–2.168	0.287

*Multi-adjusted: age, BMI, duration of infertility, embryo category, FSH, E_2_, P, PRL, LH, and T levels.

*OR odd ratio, *CI* confidence interval, *BMI* body mass index, *FSH* follicle-stimulating hormone, *E*_*2*_ estradiol, *P* progestogen, *PRL* prolactin, *LH* luteinizing hormone, *T* testosterone.

### Atosiban was beneficial to the clinical pregnancy when selecting the cleavage stage embryos for RIF

The above results suggested that atosiban could increase the clinical pregnancy rate in more than 3 ET cycles. In order to further identify the beneficiary, the subgroup analysis was performed according to the type of embryos. The results suggested that atosiban would improve the clinical pregnancy other than other pregnancy outcomes when implanting the cleavage stage embryos (D3 embryo: OR 1.721, 95% CI 1.098–2.696, *P* = 0.018). Moreover, when implanting the blastocyst, the effectiveness of atosiban for the clinical pregnancy outcomes was also disappeared (Table 4).

**Table 4:** The effects of Atosiban in the outcomes of pregnancy on for more than three embryo transfer cycles.

	Clinical pregnancy			Ectopic pregnancy			Abortion			Live birth		
	OR	95% CI	*P*	OR	95% CI	*P*	OR	95% CI	*P*	OR	95% CI	*P*
Age												
< 35 years	1.586	0.658–3.827	0.304	0.000	0.000–NA^a^	0.997	0.966	0.123–7.576	0.974	1.039	0.478–3.581	0.601
≥ 35 years	1.851	1.136–3.014	0.013	1.341E7	0.000–NA^a^	0.993	1.338	0.616–2.907	0.462	1.382	0.749–2.550	0.301
BMI												
< 24 kg/m^2^	1.522	0.934–2.481	0.092	1.737	0.216–13.956	0.604	1.813	0.770–4.270	0.173	0.994	0.549–1.802	0.985
≥ 24 kg/m^2^	2.550	1.105–5.883	0.028	NA^b^	NA	NA	0.505	0.117–2.182	0.361	6.704	1.964–22.884	0.002
Embryo												
D3	1.721	1.098–2.696	0.018	3.880	0.306–49.223	0.296	1.345	0.619–2.926	0.454	1.328	0.772–2.284	0.306
D5/D6	1.457	0.486–4.367	0.502	0.000	0.000–NA^a^	0.999	1.052	0.144–7.668	0.960	1.016	0.181–5.697	0.985

*The logistic regression analysis was performed with multi-adjusted parameters as follows: age, BMI, duration of infertility, embryo transfer cycle, embryo category, FSH, E_2_, P, PRL, LH, and T levels.

*OR odd ratio, *CI* confidence interval, *BMI* body mass index, *FSH* follicle-stimulating hormone, *E*_*2*_ estradiol, *P* progestogen, *PRL* prolactin, *LH* luteinizing hormone, *T* testosterone.

^a^The upper 95% CI is infinite. ^b^There were no samples in this outcome.

### Atosiban improved the clinical pregnancy and live birth in the obese women of RIF

When the subgroup analysis was stratified by the obesity in more than 3 ET cycles, we found that the atosiban would improve the clinical pregnancy (BMI ≥ 24 kg/m^2^: OR 2.550, 95% CI 1.105–5.883, *P* = 0.028) and the live birth in the obese women (BMI ≥ 24 kg/m^2^: OR 6.704, 95% CI 1.964–22.884, *P* = 0.002). However, these correlations were not detected for non-obese women in clinical pregnancy outcomes (Table 4).

### Atosiban promoted the clinical pregnancy in the advanced-age infertility with obesity and implanting the cleavage stage embryo

In order to further understand the factors influencing the effects of atosiban in RIF, the subgroup analysis was stratified by age. Interestingly, we found that the atosiban would improve the clinical pregnancy outcomes especially for the advanced-age (age ≥ 35 years: OR 1.851, 95% CI 1.136–3.014, *P* = 0.013) after adjusting all the confounding factors (Table 4). However, atosiban seemed not to influence the ectopic pregnancy and abortion (Table 4). Furthermore, in the RIF subgroup, atosiban application was strongly recommended for the advanced-age infertility women, who also had the risk of obesity (clinical pregnancy: OR 3.342, 95% CI 1.249–8.942, *P* = 0.016; live birth: OR 12.123, 95% CI 2.191–67.089, *P* = 0.004) and implanted the cleavage stage embryo (D3 embryo: OR 1.922, 95% CI 1.135–3.255, *P* = 0.015) (Table 5).

**Table 5:** The association between Atosiban and the clinical pregnancy and live birth in the advanced-age women with more than three embryo cycles.

	Clinical pregnancy			Live birth		
	OR	95% CI	*P*	OR	95% CI	*P*
Age ≥ 35						
BMI < 24 kg/m^2^	1.594	0.886–2.865	0.119	0.877	0.418–1.842	0.729
BMI ≥ 24 kg/m^2^	3.342	1.249–8.942	0.016	12.123	2.191–67.089	0.004
Embryo: D3	1.922	1.135–3.255	0.015	1.382	0.722–2.644	0.329
Embryo: D5/D6	2.428	0.480–12.278	0.283	3.225	0.034–3.034	0.139

*The logistic regression analysis was performed with multi-adjusted parameters as follows: age, BMI, duration of infertility, embryo transfer cycle, embryo category, FSH, E_2_, P, PRL, LH, and T levels.

*OR odd ratio, *CI* confidence interval, *BMI* body mass index, *FSH* follicle-stimulating hormone, *E*_*2*_ estradiol, *P* progestogen, *PRL* prolactin, *LH* luteinizing hormone, *T* testosterone.

## Discussion

Our study was conducted to discover the potential effect of atosiban in the pregnancy outcome especially in fresh embryo transfer cycle. The results confirmed the previous conclusion that atosiban would improve the clinical pregnancy in more than 3 ET cycles^23,25^. Moreover, the effect was more significant for the advanced-age and obese infertility women with implanting cleavage stage embryos. Our study provided clues for future studies to identify target patient population for atosiban, which would support the establishment of a more precise strategy in using atosiban among the RIFs.

IVF-ET is an advanced technology in dealing with the infertility^2,29^. Its appearance brought hope to the infertile couple. However, the success rate was not satisfactory. Successful IVF-ET was not only depended on the embryo quality, but also attributed to the intrauterine environment. It is estimated that > 30% patients would have uterine contractions to result in poor pregnancy^3–5^. In 1997, a study pointed out that abnormal endometrial peristalsis could reduce the implantation rate and clinical pregnancy rate^30^. One study had also found that frequent and strong uterine contractions 5 min after implantation could reduce the live birth rate^31^. Methods to reduce endometrial peristalsis and improve clinical pregnancy outcomes had become a hot topic in assisted reproductive technology (ART) in recent years. The other study had also found that, in the natural conception state, about 30% of embryos can be successfully implanted, while in IVF-ET, the success rate of embryo implantation was only 10–15%^32^, which might be related to the hormonal effects and ET procedure.

ET is an invasive procedure. And some studies have suggested that invasive intrauterine operations or the manipulation of the genital tract could induce the release of prostaglandins resulting in uterine contractions^31,33,34^. Then, the embryo was prevented from implanting in the uterine cavity. Pierzynski et al.^19^ also found that uterine contractions in patients with RIF were more frequent compared to the normal fertility. Ultrasound images showed that the increased frequency of intimal peristalsis and various forms of intimal movement would lead to both the difficult implantation of embryos, miscarriage of implanted embryos during development and ectopic pregnancy^19^. So, it speculated that inhibiting contractions and reducing abnormal endometrial peristaltic waves could improve pregnancy rate. And the reason of uterine contractions was attributed to the increased levels of oxytocin and PGF2a^13,14^. Oxytocin is a hormone produced by the hypothalamus and released by the posterior pituitary gland, which mainly induced the uterine contractions. And PGF2a could also reduce the perfusion of endometrial blood flow. As a safe and effective PGF2a and oxytocin-antagonists, atosiban could compete against the oxytocin receptors on uterine myocytes and inhibit the production of prostaglandin F2a (PGF2a), to reduce the uterine contractions and increase the endometrial blood supply^35^. However, some studies also identified that the positive effects of atosiban was not widespread^22^. It might only improve the IVF pregnancy for the patients with RIF or difficult transfers^36,37^. This phenomenon also confirmed in our outcomes of fresh IVF-ET. So, we suggested that atosiban would mainly increase the success rate of the IVF-ET among the patients with more than 3 ET cycles in the fresh ET.

In addition, our subgroup analysis discovered that the advanced-age women would be benefit from atosiban in the process of IVF-ET. In 2021, Buddhabunyakan et al.^38^ also found that adding atosiban in frozen embryo transfer (FET) would be advantageous for the advanced age group (women age ≥ 35 years). However, the mechanism was undefined. Recently, some studies had been focused on the effect of age in the uterine contractions. Along with the increase of age, the uterine contractions decreased after menopause^39^. Smith et al.^40^ also reported that the reduced spontaneous activity of human myometrium and increased likelihood of multiphasic spontaneous contractions happened with the increasing maternal age, which might induce the unstable intrauterine environment to influence the embryo growth and implantation. So, we speculated that prominent instability of the uterus might appear for the elderly patients while invasive intrauterine operations of IVF, although no changes of the uterine peristalsis frequency according to premenopausal age^39^. Further studies were needed to confirm this hypothesis. Additionally, in 2019, a meta-analysis suggested that female obesity was negatively associated with clinical pregnancy outcomes following IVF treatment when comparing with normal weight women^41^. Although the impacts still remained unclear in obese women, their bad uterine environment and oocyte quality might be involved in poorer reproductive outcomes^42,43^. Interestingly, our results found that atosiban would improve the clinical pregnancy outcomes of the obese women in RIF. This suggested that atosiban might improve the uterine environment in obese women by reducing uterine contractions. Meanwhile, as for the type of embryo, atosiban would be more effective in increasing the pregnancy rate for cleavage stage embryo. Studies had shown that early cleavage embryos peristalsis in the uterine cavity and fallopian tube before implantation, which leaded to a higher rate of ectopic pregnancy^44–46^. Moreover, they were more susceptible to uterine contractions than blastocysts^44–46^. Therefore, atosiban appeared to be more effective in women with cleavage embryo transfer. Additionally, atosiban had no toxicity to human sperm motility and rabbit embryo development^18,47^. And no teratogenic effect on human embryos was presented^18,47^. So, combined with our results, we proposed that atosiban was useful to improve the success rate of clinical pregnancy, especially for the advanced-age infertility and obese women while transplanting the cleavage stage embryo.

Our results might pave the way for the selection of target patient population for atosiban in the fresh embryo transfer cycle. However, some limitations also should be noticed as follows: (1) this study was a retrospective design and the results needed further validation in well-designed randomized controlled trial; (2) results of single center had limited power of promotion in the generalized population; (3) uterine contractions was not measured for patients undergoing IVF-ET.

## Conclusions

Atosiban could improve the clinical pregnancy rate and live birth rate for RIF in the fresh embryo transfer cycle of IVF-ET. In practice, atosiban would be more necessary and effective for the advanced-age and obese infertility women while implanting the cleavage stage embryo.

### Supplementary Information

Supplementary Information.

## Data Availability

Data will be made available on request from Jie Li (lijie2012@126.com) and Qiuling Shi (qshi@cqmu.edu.cn).
