# Supplementary material for: Atosiban application in fresh ET cycle is effective for women undergoing repeated embryo implantation failures, especially for advanced-age obese patients
Source: Sci Rep. 2023 Dec 27;13:23044. doi: 10.1038/s41598-023-49773-z (PMC10754826; doi:10.1038/s41598-023-49773-z)
Supplement: Supplementary file 1 — Supplementary Information. [file 41598_2023_49773_MOESM1_ESM.docx]

|  |  | Atosiban |  | Control |  | P |
| --- | --- | --- | --- | --- | --- | --- |
| No. |  | 689 |  | 15838 |  |  |
| Age |  | 35.58±4.80 |  | 34.52±4.83 |  | <0.001 |
| BMI |  | 22.35±2.66 |  | 22.16±2.82 |  | 0.085 |
| Duration of infertility |  | 4.83±3.60 |  | 4.90±3.61 |  | 0.657 |
| FSH |  | 7.88±2.82 |  | 7.76±2.74 |  | 0.241 |
| E2 |  | 43.23±26.30 |  | 45.86±30.23 |  | 0.025 |
| Progestogen |  | 0.13±1.70 |  | 0.07±1.06 |  | 0.363 |
| PRL |  | 17.14±12.62 |  | 17.07±14.93 |  | 0.906 |
| LH |  | 5.14±3.31 |  | 5.23±3.40 |  | 0.509 |
| T |  | 0.47±3.00 |  | 0.44±2.12 |  | 0.744 |
| Types of infertility |  |  |  |  |  |  |
|  | Primary | 201 |  | 4896 |  |  |
|  | Secondary | 488 |  | 10942 |  | 0.333 |
| Embryo category |  |  |  |  |  |  |
|  | D3 | 528 |  | 11757 |  |  |
|  | D5/D6 | 161 |  | 4081 |  | 0.158 |
| Embryo transfer cycle |  |  |  |  |  |  |
|  | 1 | 224 |  | 11094 |  |  |
|  | 2 | 286 |  | 3616 |  |  |
|  | ≥3 | 179 |  | 1128 |  | <0.001 |

Table S1. The characteristics of the samples before matching

* BMI: body mass index; FSH: Follicle-stimulating hormone; E2: Estradiol; P: Progestogen; PRL: Prolactin; LH: Luteinizing hormone; T: Testosterone

|  |  | Unadjusted |  |  |  | Duration of infertility-adjusted |  |  |  | Multi-Adjusted |  |
| --- | --- | --- | --- | --- | --- | --- | --- | --- | --- | --- | --- |
|  | OR | 95%CI | *P* |  | OR | 95%CI | *P* |  | OR | 95%CI | *P* |
| Age<35 years |  |  |  |  |  |  |  |  |  |  |  |
| Clinical pregnancy | 0.952 | 0.711-1.273 | 0.738 |  | 0.951 | 0.711-1.273 | 0.737 |  | 0.985 | 0.730-1.328 | 0.921 |
| Ectopic Pregnancy | 0.382 | 0.110-1.322 | 0.129 |  | 0.390 | 0.112-1.354 | 0.138 |  | 0.413 | 0.116-1.466 | 0.171 |
| Abortion | 1.230 | 0.625-2.418 | 0.549 |  | 1.233 | 0.627-2.426 | 0.543 |  | 1.288 | 0.651-2.550 | 0.467 |
| Live birth | 0.967 | 0.714-1.309 | 0.827 |  | 0.963 | 0.711-1.304 | 0.807 |  | 0.989 | 0.724-1.350 | 0.943 |
| Age≥35 years |  |  |  |  |  |  |  |  |  |  |  |
| Clinical pregnancy | 1.014 | 0.786-1.307 | 0.917 |  | 1.010 | 0.783-1.302 | 0.939 |  | 1.008 | 0.778-1.307 | 0.951 |
| Ectopic Pregnancy | 2.953 | 0.829-10.521 | 0.095 |  | 2.843 | 0.797-10.145 | 0.107 |  | 2.852 | 0.776-10.487 | 0.115 |
| Abortion | 1.021 | 0.677-1.540 | 0.920 |  | 1.011 | 0.670-1.526 | 0.957 |  | 1.008 | 0.663-1.533 | 0.970 |
| Live birth | 0.871 | 0.641-1.184 | 0.378 |  | 0.874 | 0.643-1.189 | 0.392 |  | 0.856 | 0.627-1.169 | 0.328 |

Table S2. The effects of Atosiban in the outcomes of pregnancy in different ages

*Multi-Adjusted: BMI, Duration of infertility, Embryo Transfer cycle, Embryo category, FSH, E_2_, P, PRL, LH, and T levels

* OR: odd ratio; *CI*: confidence interval; BMI: body mass index; FSH: Follicle-stimulating hormone; E_2_: Estradiol; P: Progestogen; PRL: Prolactin; LH: Luteinizing hormone; T: Testosterone

Table S3. The effects of Atosiban in the outcomes of pregnancy influenced by obesity

|  |  | Unadjusted |  |  |  | Duration of infertility-adjusted |  |  |  | Multi-Adjusted |  |
| --- | --- | --- | --- | --- | --- | --- | --- | --- | --- | --- | --- |
|  | OR | 95%CI | *P* |  | OR | 95%CI | *P* |  | OR | 95%CI | *P* |
| BMI<24kg/m^2^ |  |  |  |  |  |  |  |  |  |  |  |
| Clinical pregnancy | 0.972 | 0.779-1.212 | 0.798 |  | 0.968 | 0.775-1.207 | 0.770 |  | 0.988 | 0.786-1.242 | 0.916 |
| Ectopic Pregnancy | 0.840 | 0.321-2.199 | 0.722 |  | 0.847 | 0.323-2.219 | 0.736 |  | 0.925 | 0.349-2.454 | 0.876 |
| Abortion | 1.318 | 0.862-2.014 | 0.203 |  | 1.321 | 0.864-2.019 | 0.199 |  | 1.279 | 0.833-1.964 | 0.260 |
| Live birth | 0.883 | 0.693-1.125 | 0.314 |  | 0.877 | 0.688-1.117 | 0.287 |  | 0.891 | 0.692-1.148 | 0.373 |
| BMI≥24 kg/m^2^ |  |  |  |  |  |  |  |  |  |  |  |
| Clinical pregnancy | 0.990 | 0.686-1.427 | 0.957 |  | 0.982 | 0.680-1.417 | 0.922 |  | 0.928 | 0.631-1.364 | 0.704 |
| Ectopic Pregnancy | 1.047 | 0.259-4.234 | 0.949 |  | 1.019 | 0.251-4.127 | 0.979 |  | 1.158 | 0.250-5.355 | 0.851 |
| Abortion | 0.739 | 0.391-1.398 | 0.353 |  | 0.729 | 0.385-1.380 | 0.332 |  | 0.738 | 0.380-1.434 | 0.370 |
| Live birth | 0.981 | 0.638-1.508 | 0.930 |  | 0.980 | 0.637-1.507 | 0.926 |  | 0.884 | 0.559-1.396 | 0.596 |

*Multi-Adjusted: Age, Duration of infertility, Embryo Transfer cycle, Embryo category, FSH, E_2_, P, PRL, LH, and T levels

* OR: odd ratio; *CI*: confidence interval; BMI: body mass index; FSH: Follicle-stimulating hormone; E_2_: Estradiol; P: Progestogen; PRL: Prolactin; LH: Luteinizing hormone; T: Testosterone

Table S4. The effects of Atosiban in the outcomes of pregnancy on the basis of the embryo types

|  |  | Unadjusted |  |  |  | Duration of infertility-adjusted |  |  |  | Multi-Adjusted |  |
| --- | --- | --- | --- | --- | --- | --- | --- | --- | --- | --- | --- |
|  | OR | 95%CI | *P* |  | OR | 95%CI | P |  | OR | 95%CI | *P* |
| D3 |  |  |  |  |  |  |  |  |  |  |  |
| Clinical pregnancy | 1.056 | 0.849-1.314 | 0.623 |  | 1.046 | 0.841-1.303 | 0.684 |  | 1.049 | 0.836-1.315 | 0.680 |
| Ectopic Pregnancy | 0.992 | 0.370-2.659 | 0.988 |  | 1.009 | 0.376-2.709 | 0.985 |  | 1.063 | 0.387-2.922 | 0.905 |
| Abortion | 1.195 | 0.797-1.793 | 0.389 |  | 1.206 | 0.803-1.811 | 0.366 |  | 1.197 | 0.792-1.809 | 0.392 |
| Live birth | 0.969 | 0.759-1.236 | 0.797 |  | 0.954 | 0.747-1.218 | 0.704 |  | 0.940 | 0.729-1.212 | 0.633 |
| D5/D6 |  |  |  |  |  |  |  |  |  |  |  |
| Clinical pregnancy | 0.779 | 0.531-1.141 | 0.200 |  | 0.779 | 0.531-1.142 | 0.201 |  | 0.823 | 0.554-1.223 | 0.335 |
| Ectopic Pregnancy | 0.762 | 0.199-2.911 | 0.691 |  | 0.767 | 0.201-2.935 | 0.699 |  | 1.063 | 0.242-4.675 | 0.935 |
| Abortion | 0.823 | 0.409-1.654 | 0.584 |  | 0.836 | 0.415-1.684 | 0.617 |  | 0.790 | 0.383-1.627 | 0.522 |
| Live birth | 0.768 | 0.504-1.170 | 0.218 |  | 0.764 | 0.501-1.165 | 0.211 |  | 0.829 | 0.533-1.292 | 0.408 |

*Multi-Adjusted: Age, BMI, Duration of infertility, Embryo Transfer cycle, FSH, E_2_, P, PRL, LH, and T levels

* OR: odd ratio; CI: confidence interval; BMI: body mass index; FSH: Follicle-stimulating hormone; E_2_: Estradiol; P: Progestogen; PRL: Prolactin; LH: Luteinizing hormone; T: Testosterone
